# Supplementary material for: Characterization of Human Transition Zone Reveals a Putative Progenitor-Enriched Niche of Corneal Endothelium
Source: Cells. 2019 Oct 12;8(10):1244. doi: 10.3390/cells8101244 (PMC6829622; doi:10.3390/cells8101244)
Supplement: Supplementary file 1 [file cells-08-01244-s001.zip › Yam_Supplementary 191007.docx]

**Characterization of human transition zone reveals a putative progenitor-enriched niche of corneal endothelium**

Gary Hin-Fai Yam, Xin-Yi Seah, Nur Zahirah Binte M Yusoff, Melina Setiawan, Stephen Wahlig, Hla Myint Htoon, Gary S.L. Peh, Viridiana Kocaba, Jodhbir S Mehta

**Supplementary informataion**

**Supplementary Experimental Procedures**

**Alizarin red histochemistry** was used to visualize the cell border. Fresh human corneoscleral rims were washed thrice with OptiMEM (Invitrogen, Carlsbad, CA, USA), stained with 0.1% trypan blue solution (Sigma-Aldrich, St Louis, MO, USA) for 3 minutes, followed by minipore-filtered 0.5% Alizarin red (Sigma-Aldrich) at pH 4.5 for 3 min. After vigorous washes, the samples were flat-mounted in aqueous glycerol mounting medium (Sigma-Aldrich) and viewed under fluorescence microscopy (AxioImager II, Zeiss).

**Cell density measurement** in TZ and PE regions (within 200 µm from TZ border) (each with 8 random fields per sample; n=5 samples) and compared to central endothelial cell count provided by eye banks. The number of DAPI-stained nuclei was obtained using the count tool of Adobe Photoshop CS software (Adobe, San Jose, CA, USA) in a fixed area of 100x100 µm size and result was expressed as cell density per mm^2^.

**Immunogold scanning EM**

Human corneoscleral rims (n=3) were fixed in freshly prepared 3% paraformaldehyde /0.5% glutaraldehyde in 0.1 M sodium cacodylate buffer for 30 minutes at 4°C and cut into quadrants. After buffer washes, the samples were quenched in 0.1 M glycine in PBS for 10 minutes, followed by blocking in 1% BSA/PBS for one hour at room temperature. They were then incubated with primary mouse anti-human Lgr5 monoclonal antibody (0.5 µg/ml) for 2 hours. After multiple washes with 1% BSA/PBS, they were placed in anti-mouse IgG immunogold 15 nm (Aurion, EM Sciences) in 1% BSA/PBS at optical density 0.1 for an hour. The samples were washed and post-fixed in 2% glutaraldehyde for 10 minutes, treated for silver enhancement using R-Gent SE-EM kit (Aurion) following manufacturer’s instruction and finally washed extensively with distilled water. They were dehydrated, critical point dried, carbon-coated and viewed with back-scattered electron mode (Field Emission Scanning EM 7601/FPlus, JEOL).

**
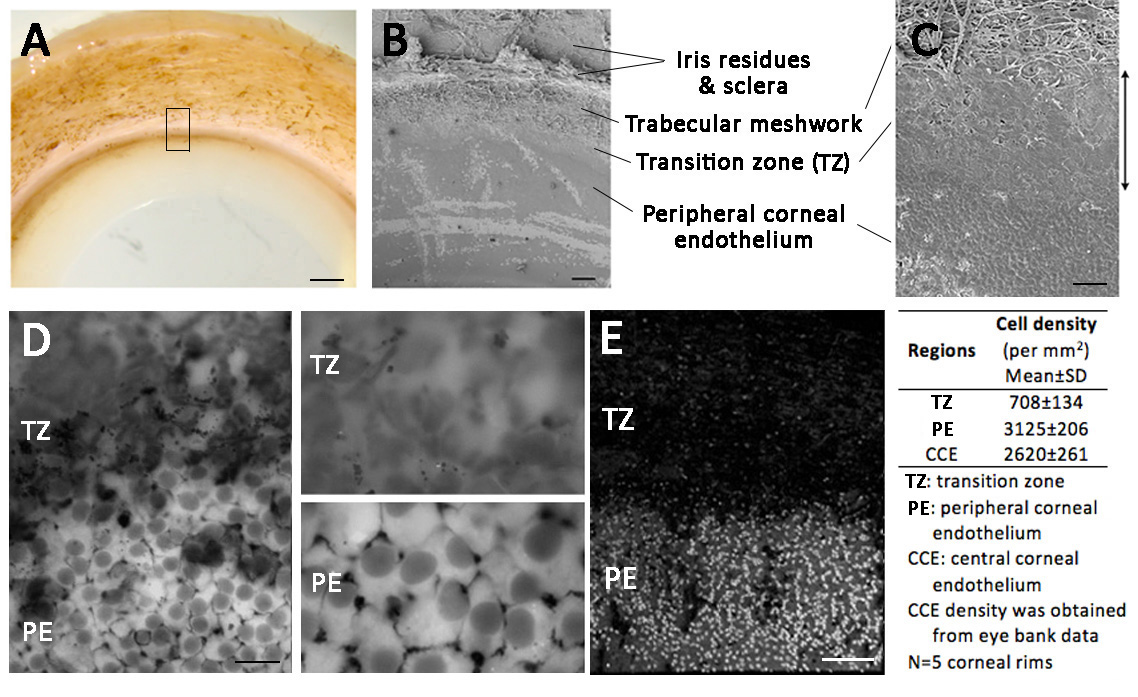
**

**Supplementary Figure S1**. **A** and **B**. Gross morphology of human posterior limbus. **A**. Stereo light micrograph. **B**. A magnified view of rectangular region in A under scanning EM. **C**. Region of transition zone (TZ) with neighboring trabecular meshwork (TM) and peripheral endothelium (PE). Arrow indicates the region of TZ. **D**. Alizarin red staining of cell border in TZ and PE. Tighter cell arrangement was found greater in PE than in TZ. **E**. Surface cell density revealed by DAPI histochemistry was significantly lower in TZ than in PE. Scale bars: 1 mm (A), 200 μm (B), 100 μm (C and E), 30 μm (D).

**
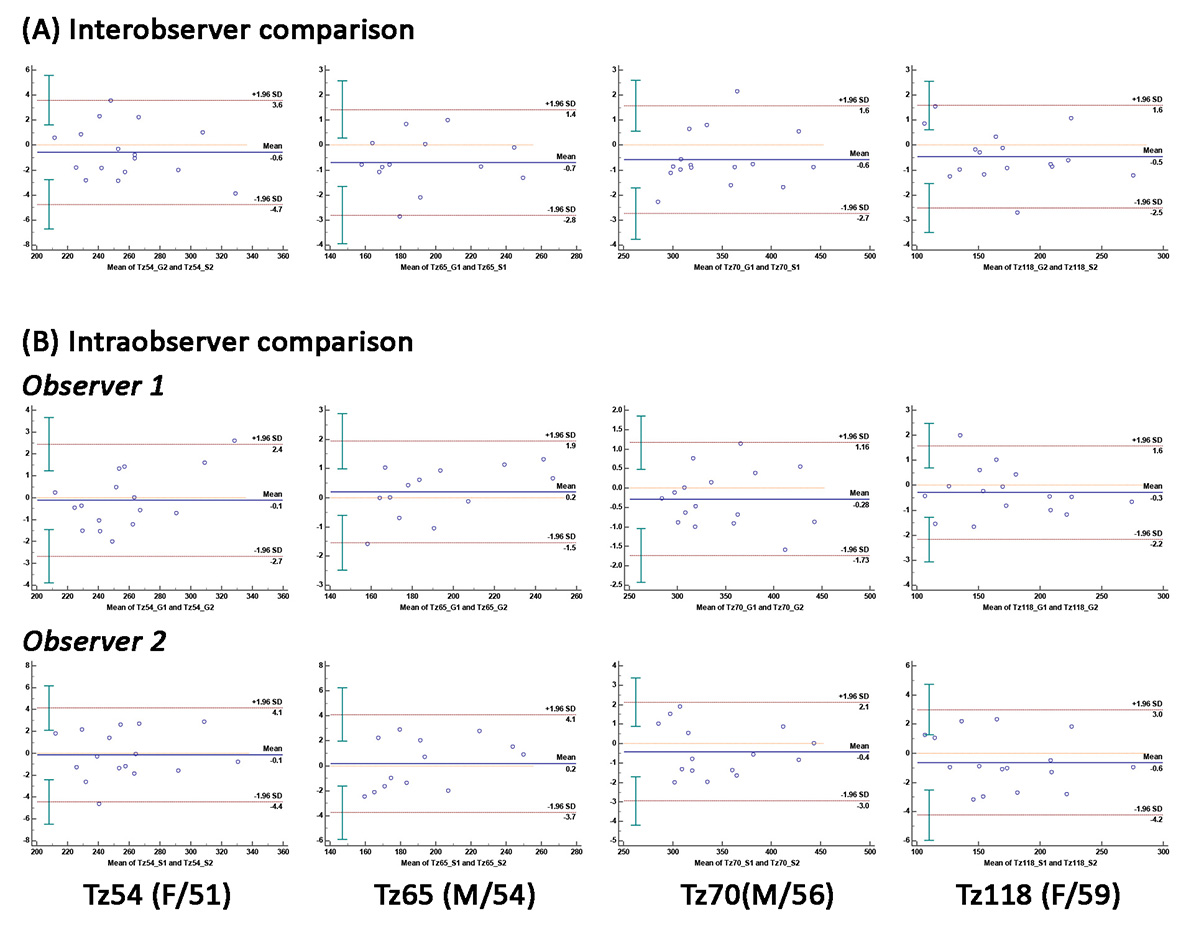
**

**Supplementary Figure S2**. Bland-Altman plots of transition zone (TZ) width of 4 human corneal samples. **A**. Inter-observer agreement. **B**. Intra-observer agreement with repeated measurement.


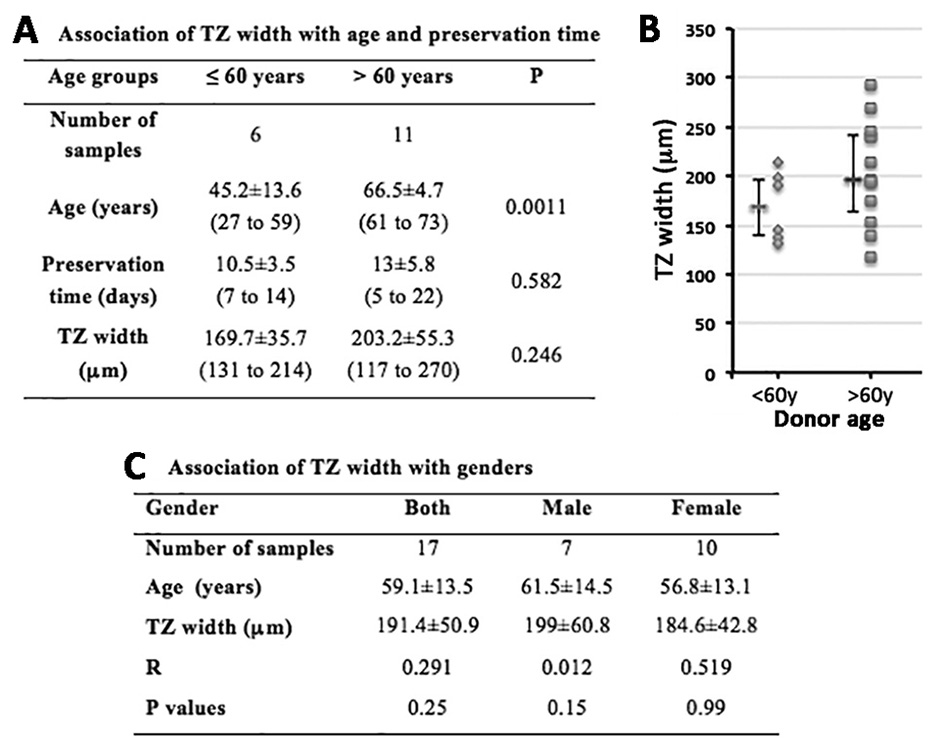


**Supplementary Figure S3**. **Demographic information and TZ width correlation analysis. A.** Table depicts the insignificant difference of mean TZ width the 2 age groups (≤60 and > 60 years old) and P=0.246 (Mann-Whitney U test). **B.** Scattered plot showing the mean TZ widths of 2 age groups. Medians and interquartile range were indicated next to the data points. **C.** Table depicts the insignificant difference of mean TZ width with genders (Spearman’st test).


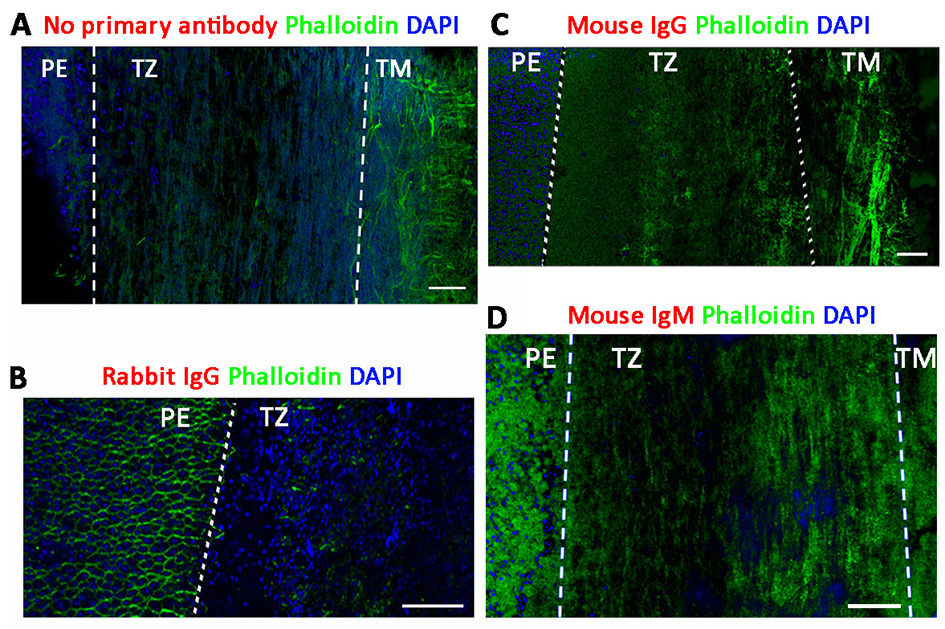


**Supplementary Figure S4. Immunostaining controls.** Z-series reconstructed confocal image of human posterior limbus showing the immunostaining (A) without primary antibody (for checking the background non-specificities), (B) rabbit IgG isotype antibody, (C) mouse IgG isotype antibody and (D) mouse IgM isotype antibody (for demonstrating the specificity of primary antibodies), together with phalloidin and DAPI. Scale bars: 50 μm.


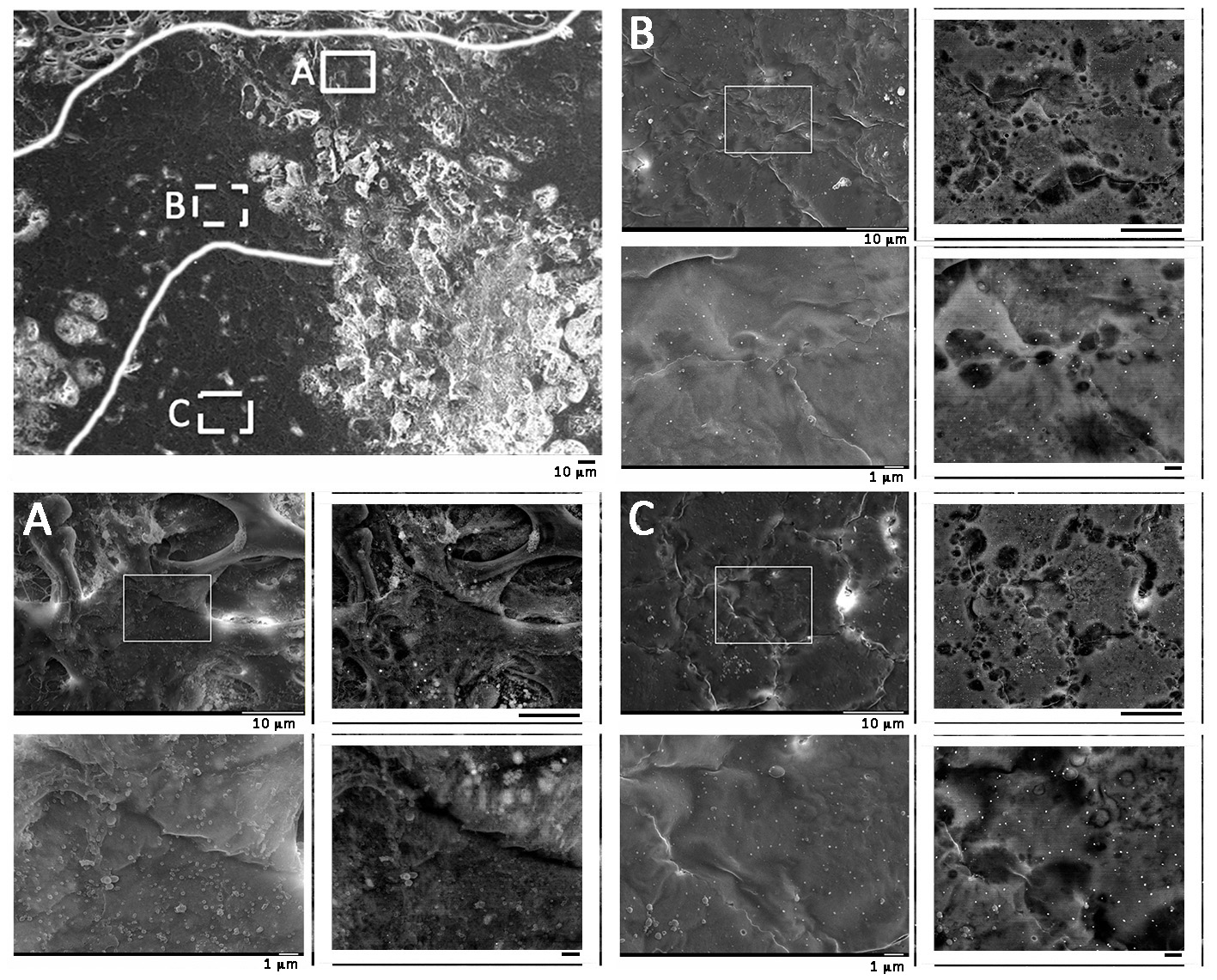


**Supplementary Figure S5. Silver-enhanced immunogold scanning electron microscopy showing regional specific expression of Lgr5.** Positive immunogold signal was confirmed by electron-opaque particles under secondary electron mode and appeared as bright particles under back-scattered mode (in bold white frame) in paired images. Selected regions in (**A**) outer TZ, (**B**) inner TZ and (**C**) peripheral endothelium (PE) were studied under serial magnification to reveal silver-enhanced gold particles representing Lgr5 signal. Positive expression was detected in inner TZ and PE, but not in outer TZ close to trabecular meshwork region.

**Supplementary Video S1. Serial block face-scanning electron microscopy (SBF-SEM) and 3D reconstruction of human transition zone/peripheral endothelium (TZ/PE) junction.** The regions of PE, TZ and TM are represented from left to right orientation. Color-coded structures are referred to Figure 8.

**Supplementary Video S2. Serial block face-scanning electron microscopy (SBF-SEM) and 3D reconstruction of human transition zone/peripheral endothelium (TZ/PE) junction.** An oblique orientation illustrated the structural transition from TM to TZ and further to PE. Color-coded structures are referred to Figure 8.

**Supplementary Table S1. Information for donor corneas.**

| **No.** | **Age** | **Sex** | **CEC count** | **Cause of death** | **Day to culture** | **Experiments** | | |
| --- | --- | --- | --- | --- | --- | --- | --- | --- |
|  |  |  | **OS / OD** |  |  | **1** | **2** | **3** |
| 1 | 24 | M | ------ / 3235 | Myocarditis | 12 | ✓ |  |  |
| 2 | 18 | M | 3536 / ------ | Subarachnoid haemorrhage | 12 | ✓ |  |  |
| 3 | 36 | F | 3199 / 3219 | Acute cardiac crisis | 10 | ✓ |  |  |
| 4 | 42 | F | ------ / 2325 | Myocardial infarction | 9 | ✓ |  |  |
| 5 | 57 | M | 2534 / ------ | Colon carcinoma | 15 | ✓ |  |  |
| 6 | 44 | M | 2146 / 2236 | Intracranial haemorrhage | 14 | ✓ |  |  |
| 7 | 54 | M | ------ / 2693 | Coronary artery disease | 12 | ✓ |  |  |
| 8 | 55 | F | 2284 / 2253 | Coronary artery disease | 14 | ✓ |  |  |
| 9 | 18 | M | ------ / 3298 | Motor vehicle accident | 12 | ✓ |  |  |
| 10 | 35 | F | 3192 / ------ | Pulmonary & cerebral edema | 16 | ✓ |  |  |
| 11 | 24 | F | 3423 / ------ | Acute cardiac crisis | 8 | ✓ |  |  |
| 12 | 31 | F | ------ / 2753 | Acute cardiac crisis | 9 | ✓ |  |  |
| 13 | 54 | F | ------ / 2585 | Stroke | 10 |  | ✓ |  |
| 14 | 48 | F | 2763 / ------ | Motor vehicle accident | 13 |  | ✓ |  |
| 15 | 38 | M | ------ / 3023 | Gastrointestinal bleeding | 13 |  | ✓ |  |
| 16 | 30 | F | ------ / 3281 | Myocarditis | 10 |  | ✓ |  |
| 17 | 47 | F | 2877 / ------ | Overdose | 11 |  | ✓ |  |
| 18 | 42 | F | 2675 / ------ | Acute cardiac crisis | 14 |  | ✓ |  |
| 19 | 38 | M | ------ / 2983 | Gastrointestinal bleeding | 9 |  | ✓ |  |
| 20 | 27 | F | ------ / 3463 | Coronary artery disease | 16 |  | ✓ |  |
| 21 | 51 | M | 2874 / ------ | Blunt trauma | 14 |  | ✓ |  |
| 22 | 72 | M | ------ / 2244 | Acute cardiac crisis | 13 |  | ✓ |  |
| 23 | 54 | M | ------ / 2706 | Acute cardiac crisis | 10 |  | ✓ |  |
| 24 | 62 | F | ------ / 2475 | Coronary artery disease | 12 |  | ✓ |  |
| 25 | 27 | M | 3215 / ------ | Stroke | 9 |  | ✓ |  |
| 26 | 59 | F | 2685 / ------ | Myocarditis | 11 |  | ✓ |  |
| 27 | 63 | F | ------ / 2563 | Motor vehicle accident | 10 |  | ✓ |  |
| 28 | 47 | F | ------ / 2902 | Gastrointestinal bleeding | 13 |  | ✓ |  |
| 29 | 61 | M | 2583 / ------ | Overdose | 14 |  | ✓ |  |
| 30 | 73 | F | ------ / 2403 | Overdose | 11 |  | ✓ |  |
| 31 | 75 | M | 2347 / ------ | Blunt trauma | 12 |  | ✓ |  |
| 32 | 63 | M | 2560 / ------ | Motor vehicle accident | 15 |  | ✓ |  |
| 33 | 51 | M | ------ / 2576 | Acute cardiac crisis | 10 |  | ✓ |  |
| 34 | 47 | F | 2896 / ------ | Gastrointestinal bleeding | 9 |  | ✓ |  |
| 35 | 70 | M | 2493 / ------ | Coronary artery disease | 8 |  | ✓ |  |
| 36 | 69 | F | 2370 / 2268 | Subarachnoid haemorrhage | 7 |  | ✓ |  |
| 37 | 66 | M | ------ / 2305 | Blunt trauma | 10 |  |  | ✓ |
| 38 | 69 | M | 1934 / 2031 | Overdose | 8 |  |  | ✓ |
| 39 | 70 | M | ------ / 2322 | Stroke | 11 |  |  | ✓ |
| 40 | 54 | F | ------ / 2687 | Sepsis | 14 |  |  | ✓ |
| 41 | 50 | F | 2254 / 2269 | Gastrointestinal bleeding | 10 |  |  | ✓ |
| 42 | 36 | F | ------ / 3023 | Myocarditis | 11 |  |  | ✓ |
| 43 | 39 | M | ------ / 3343 | Acute cardiac crisis | 15 |  |  | ✓ |
| 44 | 48 | M | 2984 / ------ | Overdose | 9 |  |  | ✓ |
| 45 | 51 | F | ------ / 2788 | Coronary artery disease | 9 |  |  | ✓ |
| 46 | 76 | F | ------ / 2284 | Colon carcinoma | 10 |  |  | ✓ |
| 47 | 59 | M | 2573 / ------ | Myocarditis | 14 |  |  | ✓ |

Donor age ranged from 18 to 76 years old with median 51 years old, 49.44±15.76. F/M: 24/23. Day to culture: time of Optisol preservation prior to primary cell isolation and culture. ✓ Usage in experiments for

1. Immunostaining (whole mounts, sections)
2. Electron microscopy (scanning, transmission, SBF-SEM, immunogold SEM)
3. RNA analysis

Abbreviations: F: female; M: male; OS, ocular sinister (left eye); OD, ocular dexter (right eye); CEC, corneal endothelial cell.

**Supplementary Table S2. Primary antibody information**

| **Antibody (clone)** | **Isotype** | **Company**  **(catalogue no.)** | **Applications** |
| --- | --- | --- | --- |
| ABCG2 (5D3) | Mouse IgG2b | Millipore (MAB4155) | IF: 2 μg/ml |
| ACTB-horseradish peroxidase (AC-15) | Mouse IgG1 | Sigma-Aldrich (A3854) | 1:20,000 dilution |
| ALDH3A1 | Rabbit IgG | Proteintech (15578-1) | IF: 2 μg/ml |
| CD34 (QBEND/10) | Mouse IgG1 | Millipore (CBL496) | IF: 2 μg/ml |
| CD90/Thy1 (5E10) | Mouse IgG1 | BD (550402) | IF: 1 μg/ml |
| HNK1/CD57 (VC1.1) | Mouse IgM | Sigma-Aldrich (C6680) | IF: 1 μg/ml |
| Lgr5 (OTI2A2) | Mouse IgG1 | Origene (TA503316) | IF: 1 μg/ml Im-SEM: 2 μg/ml |
| Na^+^K^+^ATPase (H-3) | Mouse IgG2b | SantaCruz Biotech (sc-48345) | IF: 2 μg/ml WB: 0.5 μg/ml |
| Nestin (10c2) | Mouse IgG1 | SantaCruz Biotech (sc23927) | IF: 0.5 μg/ml |
| P75NTR (ME20.4) | Rabbit IgG | Millipore (AB1554) | IF: 2 μg/ml |
| Pitx2 ABC | Rabbit IgG | Capra (PA1020) | IF: 1 μg/ml |
| Prdx6 (TAG-2A12) | Mouse IgG | Raised in-house (Ding et al., 2014) | IF: 2 μg/ml WB: 1 μg/ml |
| Telomerase (2C4) | Mouse IgM | ThermoFisher (MA5-16034) | IF: 2 μg/ml |
| Vimentin (RV202) | Mouse IgG | Abcam (ab8978) | IF: 1.5 μg/ml |
| ZO-1 (R40.76) | Rabbit IgG | Millipore | WB: 1 μg/ml |
| Phalloidin-AlexaFluor 488 |  | Invitrogen (A12379) | IF: 1:600 dilution |
| Mouse IgG isotype control |  | SantaCruz (sc-2025) | IF: 1:500 |
| Mouse IgM isotype control |  | SantaCruz (sc-3881) | IF: 1:500 |
| Rabbit IgG isotype control |  | Invitrogen (08-6199) | IF: 1:500 |

* IF: immunofluorescence; Im-SEM: immuno-scanning EM; WB: western blotting

**Supplementary Table S3. Expression primers**

|  | **Gene** | **GeneBank Accession No.** | |  |
| --- | --- | --- | --- | --- |
| Oligonucleotide primers | | | | |
| 1 | COL8A2 | NM_005202.2 | F: ACATCCAGCCCATGCAGAAA  R: GCATTTCCAGGTACTGGCCT | |
| 2 | GAPDH | NM_002046.6 | F: TGTGGTCATGAGTCCTTCCA  R: CGAGATCCCTCCAAAATCAA | |
| 3 | P75NTR | - NM_002507.3 | F: CCTACGGCTACTACCAGGATG  R: CACACGGTGTTCTGCTTGTC | |
| 4 | SNAIL1 | NM_005985.3 | F: GACCCCAATCGGAAGCCTAACT  R: AGCCTTTCCCACTGTCCTCATCT | |
| 5 | SOX9 | - NM_000346.3 | F: TTCACCTACATGAACCCCGC  R: CAAGGTCGAGTGAGCTGTGT | |
| 6 | ZO1 | - NM_003257.3 | F: GTCCAGAATCTCGGAAAAGTGC  R: CTTTCAGCGCACCATACCAACC | |
| Taqman assay IDs | | | | |
| 7 | ABCG2 | NM_001257386 | Hs01053790_m1 | |
| 8 | ACTB | NM_0011001.4 | Hs01060665_g1 | |
| 9 | FOXC1 | - NM_001453.3 | Hs00559473_s1 | |
| 10 | KLF4 | - NM_001314052.1 | Hs00358836_m1 | |
| 11 | C-MYC | NM_002467.5 | Hs00153408_m1 | |
| 12 | NANOG | NM_024865.4 | Hs02387400_g1 | |
| 13 | OCT3/4 | - NM_002701.5 | Hs04260367_gH | |
| 14 | PITX2 | - NM_000325.5 | Hs04234069_mH | |
| 15 | SLC4A11 | - NM_032034.3 | Hs00984691_g1 | |
